# dbAMP 2.0: updated resource for antimicrobial peptides with an enhanced scanning method for genomic and proteomic data

**DOI:** 10.1093/nar/gkab1080

**Published:** 2021-11-29

**Authors:** Jhih-Hua Jhong, Lantian Yao, Yuxuan Pang, Zhongyan Li, Chia-Ru Chung, Rulan Wang, Shangfu Li, Wenshuo Li, Mengqi Luo, Renfei Ma, Yuqi Huang, Xiaoning Zhu, Jiahong Zhang, Hexiang Feng, Qifan Cheng, Chunxuan Wang, Kun Xi, Li-Ching Wu, Tzu-Hao Chang, Jorng-Tzong Horng, Lizhe Zhu, Ying-Chih Chiang, Zhuo Wang, Tzong-Yi Lee

**Affiliations:** Warshel Institute for Computational Biology, The Chinese University of Hong Kong, Shenzhen 518172, China; Warshel Institute for Computational Biology, The Chinese University of Hong Kong, Shenzhen 518172, China; School of Science and Engineering, The Chinese University of Hong Kong, Shenzhen 518172, China; Warshel Institute for Computational Biology, The Chinese University of Hong Kong, Shenzhen 518172, China; School of Science and Engineering, The Chinese University of Hong Kong, Shenzhen 518172, China; Warshel Institute for Computational Biology, The Chinese University of Hong Kong, Shenzhen 518172, China; School of Life and Health Sciences, The Chinese University of Hong Kong, Shenzhen 518172, China; Department of Computer Science and Information Engineering, National Central University, Taoyuan 32001, Taiwan; Warshel Institute for Computational Biology, The Chinese University of Hong Kong, Shenzhen 518172, China; School of Science and Engineering, The Chinese University of Hong Kong, Shenzhen 518172, China; Warshel Institute for Computational Biology, The Chinese University of Hong Kong, Shenzhen 518172, China; Warshel Institute for Computational Biology, The Chinese University of Hong Kong, Shenzhen 518172, China; School of Science and Engineering, The Chinese University of Hong Kong, Shenzhen 518172, China; Warshel Institute for Computational Biology, The Chinese University of Hong Kong, Shenzhen 518172, China; Warshel Institute for Computational Biology, The Chinese University of Hong Kong, Shenzhen 518172, China; School of Life and Health Sciences, The Chinese University of Hong Kong, Shenzhen 518172, China; School of Life and Health Sciences, The Chinese University of Hong Kong, Shenzhen 518172, China; School of Life and Health Sciences, The Chinese University of Hong Kong, Shenzhen 518172, China; School of Life and Health Sciences, The Chinese University of Hong Kong, Shenzhen 518172, China; School of Life and Health Sciences, The Chinese University of Hong Kong, Shenzhen 518172, China; Warshel Institute for Computational Biology, The Chinese University of Hong Kong, Shenzhen 518172, China; Warshel Institute for Computational Biology, The Chinese University of Hong Kong, Shenzhen 518172, China; Department of Biomedical Sciences and Engineering, National Central University, Taoyuan 32001, Taiwan; Graduate Institute of Biomedical Informatics, Taipei Medical University, Taipei 10675, Taiwan; Department of Computer Science and Information Engineering, National Central University, Taoyuan 32001, Taiwan; Warshel Institute for Computational Biology, The Chinese University of Hong Kong, Shenzhen 518172, China; School of Life and Health Sciences, The Chinese University of Hong Kong, Shenzhen 518172, China; School of Life and Health Sciences, The Chinese University of Hong Kong, Shenzhen 518172, China; Warshel Institute for Computational Biology, The Chinese University of Hong Kong, Shenzhen 518172, China; Warshel Institute for Computational Biology, The Chinese University of Hong Kong, Shenzhen 518172, China; School of Life and Health Sciences, The Chinese University of Hong Kong, Shenzhen 518172, China

## Abstract

The last 18 months, or more, have seen a profound shift in our global experience, with many of us navigating a once-in-100-year pandemic. To date, COVID-19 remains a life-threatening pandemic with little to no targeted therapeutic recourse. The discovery of novel antiviral agents, such as vaccines and drugs, can provide therapeutic solutions to save human beings from severe infections; however, there is no specifically effective antiviral treatment confirmed for now. Thus, great attention has been paid to the use of natural or artificial antimicrobial peptides (AMPs) as these compounds are widely regarded as promising solutions for the treatment of harmful microorganisms. Given the biological significance of AMPs, it was obvious that there was a significant need for a single platform for identifying and engaging with AMP data. This led to the creation of the dbAMP platform that provides comprehensive information about AMPs and facilitates their investigation and analysis. To date, the dbAMP has accumulated 26 447 AMPs and 2262 antimicrobial proteins from 3044 organisms using both database integration and manual curation of >4579 articles. In addition, dbAMP facilitates the evaluation of AMP structures using I-TASSER for automated protein structure prediction and structure-based functional annotation, providing predictive structure information for clinical drug development. Next-generation sequencing (NGS) and third-generation sequencing have been applied to generate large-scale sequencing reads from various environments, enabling greatly improved analysis of genome structure. In this update, we launch an efficient online tool that can effectively identify AMPs from genome/metagenome and proteome data of all species in a short period. In conclusion, these improvements promote the dbAMP as one of the most abundant and comprehensively annotated resources for AMPs. The updated dbAMP is now freely accessible at http://awi.cuhk.edu.cn/dbAMP.

## INTRODUCTION

The abuse of traditional antibiotics has resulted in the development of widespread bacterial drug resistance, which can cause serious health problems worldwide (1). It is also becoming increasingly difficult to identify new antibiotics, making the search for alternatives even more important. Antimicrobial peptides (AMPs) are a class of peptides composed of cationic and hydrophobic amino acids with direct antibacterial activity (2). AMPs range in size from <10 to hundreds of amino acids. They are an important part of the innate immune system acting to protect the host from various pathogens and viruses (3,4). These cationic AMPs bind and interact with negatively charged bacterial cell membranes, resulting in changes in their electrochemical potential, which induces cell membrane damage and allows for penetration of larger molecules, such as proteins, destroying cell morphology and ultimately leading to cell death. These AMPs have been proven to have several advantages over traditional antibiotics and exhibit broad-spectrum antimicrobial activities, including antibacterial, antifungal, antiviral and anticancer activities, with some AMPs even able to overcome acquired drug resistance (5). Additionally, the COVID-19 pandemic is unlikely to end until there is a global rollout of treatment that protects against severe disease and drives herd immunity. The COVID-19 yields a severe threat to human health with a high transmission rate, critical symptoms and relatively high mortality rate in some areas. Thus, there is an urgent need to search for effective therapeutic agents targeting the virus. AMPs are widely recognized as promising solutions for harmful microorganisms (2) making them an active target for the development of novel anti-SARS-CoV-2 therapies. Despite this, there have been relatively few descriptions of AMPs or antiviral peptides (AVPs) with any documented antiviral effect. Interestingly, there are a handful of reports describing prophylactic effects for some AVPs used in the treatments of other coronaviruses (6,7). This includes a paper by Zhao *et al.* (8), which showed that a short peptide, called P9, had robust antiviral effects against a variety of respiratory viruses *in vitro* and *in vivo*, including influenza A virus (H1N1, H3N2, H5N1, H7N7 and H7N9), SARS-CoV and MERS-CoV (https://awi.cuhk.edu.cn/dbAMP/information.php?db=dbAMP_19909). In addition to these peptides, various studies have shown that AMPs from amphibian skin, such as caerin (9) and temporin (10–12), have antiviral activities. A recent *in silico* study by Liscano *et al.* indicated that two amphibian AMPs, caerin 1.6 and caerin 1.10, had a high affinity for the spike protein of SARS-CoV-2 (13). These results greatly encouraged our expectations that AMPs could be used as alternative drugs in the treatment of COVID-19. Moreover, by failing to address the escalating antimicrobial resistance (AMR) issue, the near-complete beginning of the post-antibiotic era could lead to more infectious deaths and global financial uncertainty by 2050 (14–17). AMPs are a novel class of alternatives that possess potent activity against a wide range of Gram-negative bacteria with little or no capacity to induce AMR (16). This has stimulated the substantial development of new peptide-based antibiotics with improved therapeutic indices (18).

With the fast growing number of AMPs, it becomes challengeable to handle the large quantity of data manually. Therefore, it is of great help to build databases focused on AMPs. Over the last decade, many AMP-related databases have been established to support AMP deposition, query and mining, as a means to develop computational tools for AMP prediction and design. These resources may be separated into two main groups: general and specific databases. For those databases that focus on the collection of general AMPs, the Antimicrobial Peptide Database, established in 2004, is the most popular one, and it acts as a repository for natural AMPs and includes >3200 compounds from a wide variety of organisms (19). Another resource for general AMPs is CAMPR3, which provides AMP family identification based on signature sequences and structural folds, which can help identify key elements during antimicrobial drug design (20). Some other databases for general AMPs, such as DRAMP (21) and LAMP (22), were designed to provide patented peptides and cross-links with other AMP databases, respectively. There was also a recent update to DBAASP, which continues to develop novel prediction tools for the *de novo* design of peptide-based drugs (23). In addition, many studies have provided experimental data describing the efficacy of various peptide-based antimicrobial agents against Gram-positive or Gram-negative bacteria (24) and some works focused on AMPs with specific functional activities. The databases for antiviral peptides [AVPdb (25)], defensins knowledgebase (26), synthetic peptides [SAPD (27)] and recombinantly produced AMPs [RAPD (28)] were designed to capture these data. There are also other specialized databases, such as CancerPPD, Hemolytik, THPdb, InverPep and AntiTbPdb, which were designed to facilitate the curation of field-specific data. The CancerPPD (29) database describes anticancer peptides and proteins, the Hemolytik (30) database curates data around experimentally confirmed hemolytic and nonhemolytic peptides, THPdb (31) supplies information on FDA-approved peptide and protein therapeutics, InverPep (32) describes the AMPs from invertebrates and the AntiTbPdb (33) includes a description of the experimentally verified antitubercular or antimycobacterial peptides.

The dbAMP launched its first manually annotated AMP data storage in 2018 (34), focusing on collecting natural and synthetic AMPs and providing general, structural and >20 types of functional activities linked to published works. In addition, given the wide application of next-generation sequencing (NGS) and third-generation sequencing, the dbAMP was also designed to provide a platform for AMP exploration and functional prediction supported by *in silico* determination of critical physicochemical properties from high-throughput data. Here, we describe the updates and new features in the dbAMP platform, which may serve as a helpful resource for AMP study and design. dbAMP 2.0 provides a homology-based gene prediction program, an integrated tool stream that combines open reading frame (ORF) prediction and AMP classification to identify probably AMPs directly from genome or proteome sequencing data. Considering the rapid development of computational tools, it is expected that highly accurate prediction models could help researchers improve scoring functions for the design and prediction of AMP sequences while reducing their development costs. These updates move us toward a more harmonized system for AMP production and provide a powerful unified source for initial AMP investigation.

## SYSTEM OVERVIEW AND DATABASE UPDATES

### Updated database content and data statistics

**Figure 1.**
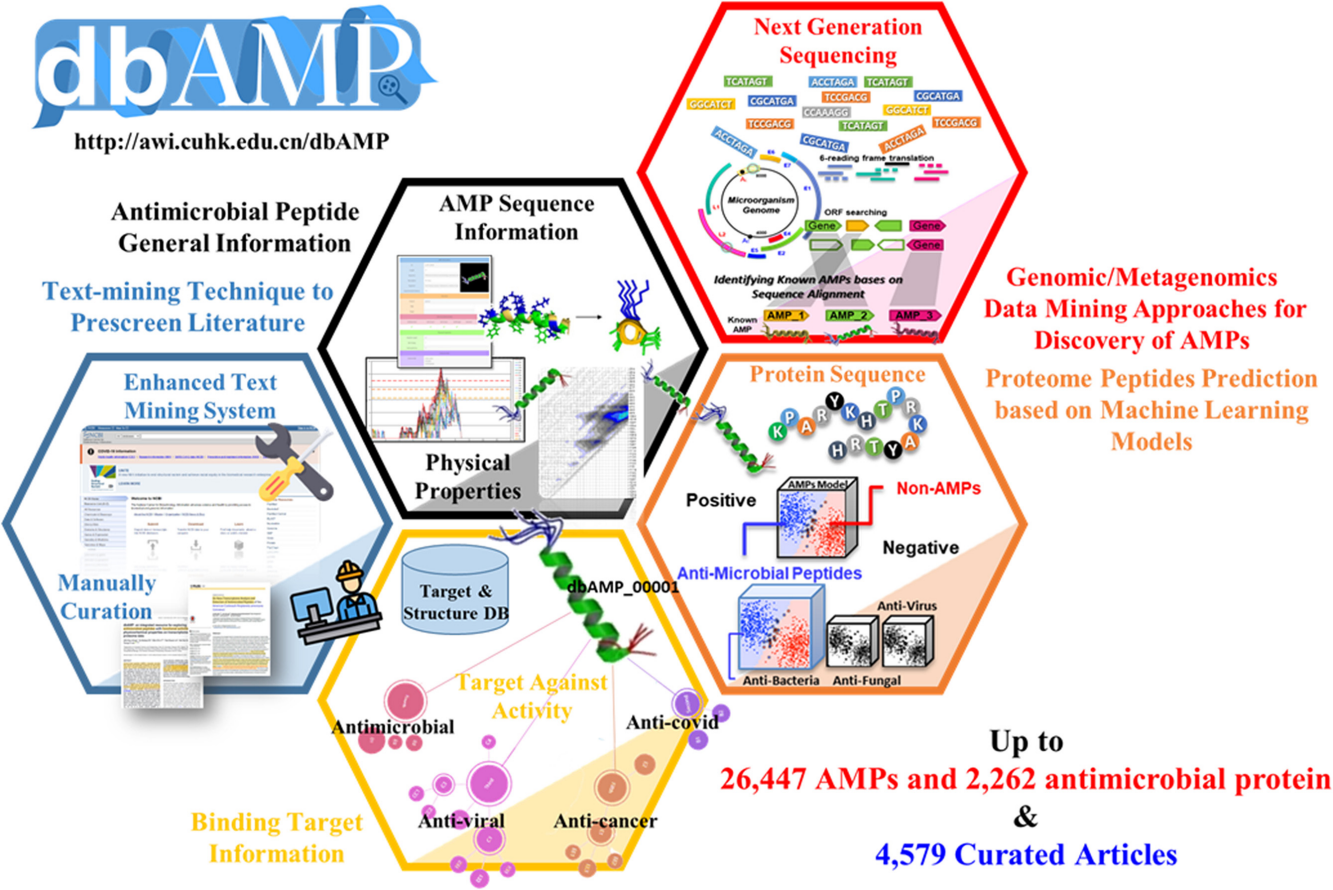
Highlighted improvements in dbAMP 2.0. dbAMP is the most comprehensive resource for AMPs with this update bringing the total values for the AMP sequences and curated articles to >28 000 and >4500, respectively.

Since the first version of dbAMP (34) was released in 2018, the number of natural and artificial AMPs has increased drastically, along with the attention of global research groups to drug resistance issues. Table 1 describes the improvements and updated content in dbAMP 2.0. These improvements include an increase in the number of curated articles, AMP sequences and source organisms. Additionally, to extract useful information from the curated articles, a new text mining system was built to enhance the automated recognition of AMP-related articles through a scoring system. Specifically, natural language processing was adopted to extract needed information from articles. The extracted information was then integrated into the updated database. As of June 2021, this update had significantly increased the number of AMPs, >2-fold when compared with dbAMPv1, and included a total of 9454 AMP–target interactions between 28 709 entries (including 26 447 AMPs and 2262 antimicrobial proteins) and 5531 target organisms as described in 4579 research articles (Figure 1). The distribution of the AMP source organisms is shown in Supplementary Figure S1 with the most common source organisms being the amphibia (28.1%), mammals (22.1%), arthropods (11.1%) and Viridiplantae (10.1%). In addition, literature and related database records allowed for the functional characterization of these AMPs within the dbAMP categorizing the AMP data into eight major functional classes with 53 functional activities. The most populated classes were the antibacterial peptides (68.33%), followed by new functional peptides (30.88%), antifungal peptides (19.29%), disease-associated peptides (11.48%), antiviral peptides (6.2%), antiparasitic peptides (1.43%), toxic peptides (0.78%) and new mechanism-associated peptides (0.63%) (Table 2). It is of interest to note that this update included over 180 anticoronavirus peptides, with experimental validation.

**Table 1.** Comparison between this update and existing AMP databases

Features	dbAMP	LAMPv2	DBAASPv3	DRAMPv2	dbAMP 2.0
**Release date (latest)**	January 2019	March 2020	November 2020	May 2021	June 2021
**Number of AMPs**	12 389	23 253	>15 700	22 151	26 447 AMPs and 2,262 antimicrobial proteins
**Organisms**	2048	–	–	–	3044
**Tertiary structures (PDB structure)**	1169	–	>3600 (molecular dynamic trajectory >3200)	283	3444
**Tertiary structures (predicted structure)**	–	–	–	263	458
**Number of biological activities**	26	38	–	11	53
**Number of target organisms**	1737	–	6560	–	5531
**Curated AMP–target interactions**	6338	–	–	–	9454
**Text mining technique to prescreen literature**	Text extraction system	–	–	–	Enhanced NLP system
**Download dataset**	Yes	Yes	Yes	Yes	Yes
**Benchmark datasets for prediction**	Yes	–	–	–	Yes
**Application utilities**					
**Antimicrobial potency analysis**	Yes	–	–	–	Yes
**Detection of cryptic region in AMPs**	Yes	–	–	–	Yes
**AMP prediction**	AMP prediction models based on multiple species	–	Prediction of general antimicrobial activity/against activity	–	*AMPpredictor*: enhance AMP prediction models
**NGS data analysis**	AMP sequence alignment based on Bowtie2	–	–	–	*AMPfinder*: genomic/proteomic data mining approaches for the discovery of AMPs

The terms that could not be identified or missing are recorded as ‘–’.

**Table 2.** Comparison of the data statistics from this update and the previous version in terms of functional activities

Function classes	Against activity	dbAMP	dbAMP 2.0^a^ (%)
Antibacterial	Antibacterial	3006	4837 (18.29)
	Anti-Gram-positive	2726	11 652 (44.06)
	Anti-Gram-negative	2323	12 405 (46.91)
	Antimicrobial	4816	8654 (32.72)
	Antibiofilm	40	40 (0.14)
	Mollicute	–	36 (0.14)
	Antiyeast	4	5 (0.02)
	Antilisterial	–	2 (0.01)
Antifungal	Antifungal	1623	5454 (20.62)
Antiviral	Antiviral	300	1745 (6.60)
	Anti-SARS/CoV	–	186 (0.70)
Antiparasitic	Antiparasitic	123	186 (0.70)
New function peptides	Mammalian cells	308	402 (1.52)
	Anuran defense	–	7256 (27.44)
	Insecticidal	35	1791 (6.77)
	Antiprotozoal	6	195 (0.74)
	Chemotactic	59	61 (0.23)
	Antimalarial	26	46 (0.17)
	Antinematode	–	46 (0.17)
	Antiplasmodial	–	35 (0.13)
	Cell penetrating	–	29 (0.11)
	Enzyme inhibitor	26	26 (0.10)
	Wound healing	19	21 (0.07)
	Antibiotic	–	19 (0.07)
	Immunomodulant	–	17 (0.06)
	Spermicidal	13	13 (0.05)
	Edema inducer	–	11 (0.04)
Disease-associated peptides	Anticancer	227	2290 (7.98)
	Anti-HIV	109	2286 (8.64)
	Antitumor	9	1018 (3.85)
	Anti-HCV	–	67 (0.25)
	Antiangiogenesis	–	13 (0.05)
	Anti-HSV	–	10 (0.04)
	Antiallodynic	–	1 (0.01)
New mechanism-associated peptides	Antihypertensive	–	1 (0.01)
	Anti-MRSA	–	874 (3.30)
	Antidiabetic	–	113 (0.43)
	Antioxidant	22	31 (0.12)
	Surface immobilized	19	27 (0.10)
	Mast cell degranulating	–	18 (0.07)
	Uterotonic	–	6 (0.02)
	Anti-inflammatory	–	6 (0.02)
	Antineurotensive	–	4 (0.02)
	Plasma anticlotting	–	3 (0.01)
	Proteolytic	–	2 (0.01)
	Antinociceptive	–	2 (0.01)
	Hypotensive	–	1 (0.01)
	Sodium channel blocker	2	2 (0.01)
Toxic	Cytotoxin	–	1 (0.01)
	Hemolytic^a^	–	115 (0.43)
	Cytolytic	–	98 (0.37)
	Ichthyotoxic	–	14 (0.05)
			

^a^ The numbers in parentheses are displayed as theproportion of entries in the dbAMP.

### 3D structure visualization of AMPs

**Figure 2.**
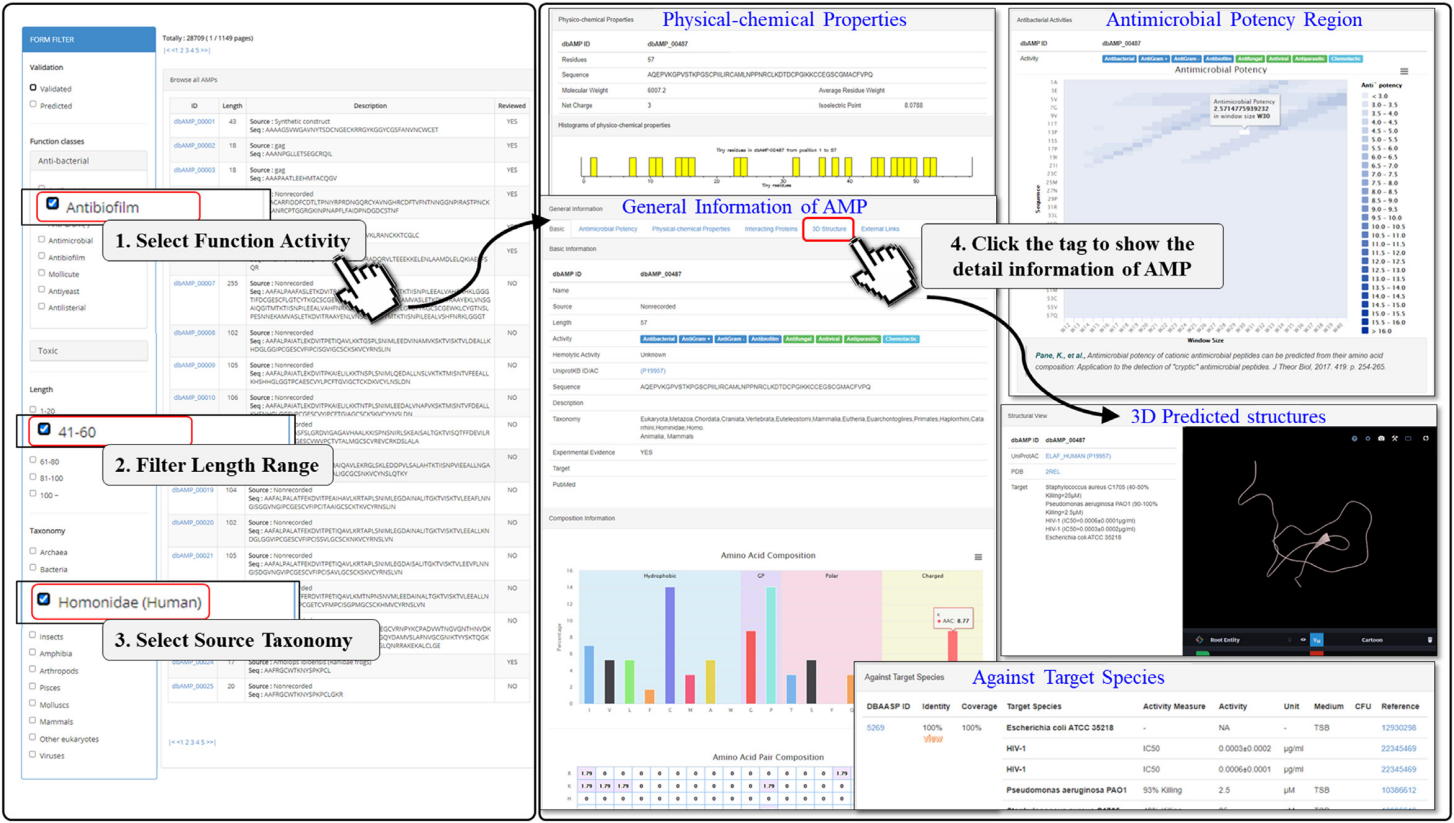
The predicted structure viewer was integrated into the platform during this update. A case study describing the production of AMP, elafin (dbAMP_00487), which is the major antiviral protein in cervicovaginal lavage fluid, using human γδ T cells.

AMPs have experienced a resurgence in interest resulting from the increasingly serious problem of antibiotic resistance. These peptides have attracted significant attention as potential therapeutic agents because they combine the high selectivity, potency and advantages of biological agents with the low toxicity, conformational limitation and cost reductions (35–38). Natural AMPs have been applied in traditional medicine and appear to be reasonable choices for clinical trials and practical applications (35,39). However, the structural characteristics of these peptides are often unstable, and their pharmacokinetic characteristics are poorly described, which seriously hinders their further application as drugs (40). Scientists hope to find an alternative to antibiotics as soon as possible. So far, we have collected 2442 validated AMP structures with 100% sequence identities by alignment from PDB. Meanwhile, 1002 validated structures of AMPs are matched with the criteria of sequence identities ≥90% and *E*-values ≤10^–5^. After the sequence alignment, totally 1059 AMPs can be mapped onto the 3444 entries of PDB (Table 1). Moreover, certain AMPs [e.g. segments from most plants (41) or histidine-rich human histatin (42)] are nested on their parent proteins. These AMPs are consequently extracted and isolated on demand (43). As a matter of fact, the isolated segments of the parent proteins possess different structures from the crystallization of their sources. Therefore, to provide complete AMP structure information and accelerate the development of these therapeutic drugs, there is an urgent need to combine computational methods with classical functional evaluation to provide a streamlined approach to novel antimicrobial development. The use of simulated structural evaluations would allow for high-throughput screening and a more robust hit ratio for downstream development. Due to a lack of validated 3D structures for these stapled peptides, structural prediction may be an alternative way to realize structure visualization (44). In this update, the 3D structure for each AMP without experimentally confirmed PDB entries was predicted using I-TASSER allowing for automated protein structure prediction and structure-based functional annotation (45). The I-TASSER server is an online platform for protein structure and function predictions that can produce novel structural predictions using known structures or *ab initio* using sequence data alone. Thus, we retrieved the relevant structural templates from PDB using the multithread splitting method and then constructed a novel structure prediction model using segment assembly simulation. We then matched the predicted structural model to known proteins in the functional database and added the relevant functional information. Until now, there are 458 3D structures for the current entries of the database (Table 1) that have been developed by this tool and are available for the further utilizations. Figure 2 reveals that dbAMP 2.0 can provide comprehensive functional analysis and predicted structures for each peptide in the ‘3D structure’ information pages.

### A systematic pipeline for the discovery of AMPs on genomic and transcriptomic data

**Figure 3.**
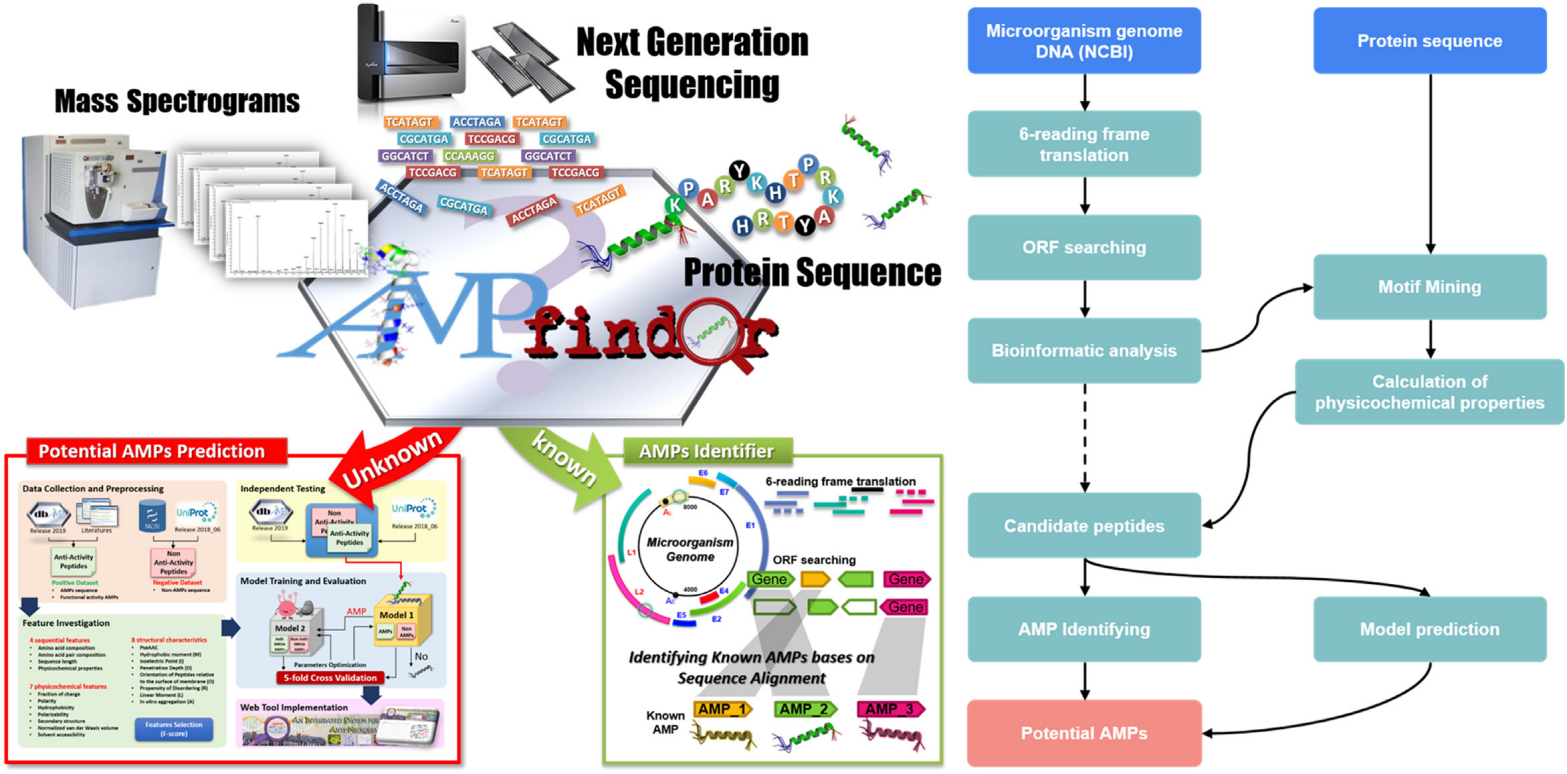
Main pipeline workflow for AMPfinder. AMPfinder is an efficient online tool, which can accurately identify AMPs within genome/transcriptome and proteome data in a short period of time.

AMPfinder is a simple, yet accurate, computational pipeline that processes either whole genome/metagenome or proteome sequences, and combines ORF prediction with accurate AMP classification to facilitate AMP prediction from protein or nucleotide data. The search for AMPs is based on alignment searching the existing AMP databases and predicting the feature model from the amino acid sequences obtained from the translation of the original transcriptome sequence data (Figure 3). AMPfinder provides a powerful alignment tool for both DNA and protein sequences using the data available in dbAMP. Prodigal is a free, open-source bioinformatics-based algorithm that efficiently predicts protein-coding genes (46). AMPfinder predicts ORFs using Prodigal when queried using a DNA sequence, which translates the input transcriptome data and selects short sequences containing ORFs and signal peptide cleavage sites. If protein sequences are submitted, AMPfinder will skip the prediction of ORFs and directly use these protein sequences. Then, BLAST (47) (for the command-line tool) or Diamond (48) (for dbAMP website) was used for homology detection and machine learning prediction model for the search of potential AMPs, in which case all known or potential motifs will be revealed and classified. AMPfinder used the AMP prediction module built in the first release of dbAMP. The training set was adapted from the dataset proposed by Wang, Hu and coworkers (49,50). After removing redundant sequences, the training set (containing 2399 AMPs and 26 850 non-AMPs with a ratio of ∼1:10) was classified into seven common species according to their source organisms. As reported by Chung *et al.* (51), the random forest was the best classifier for predicting AMPs in these seven categories of organisms. The accuracies of all the predictive models were >93% (Supplementary Table S1). With the promising performance in predicting AMPs on microorganisms, AMPfinder can effectively identify AMPs throughout large-scale genome sequences of all species, whereas current general-purpose gene prediction programs mainly focus on specific species (52,53). Therefore, AMPfinder could be an efficient and effective tool for the rapid screening of potential AMPs.

### Enhanced prediction of AMPs using proteomic data

**Figure 4.**
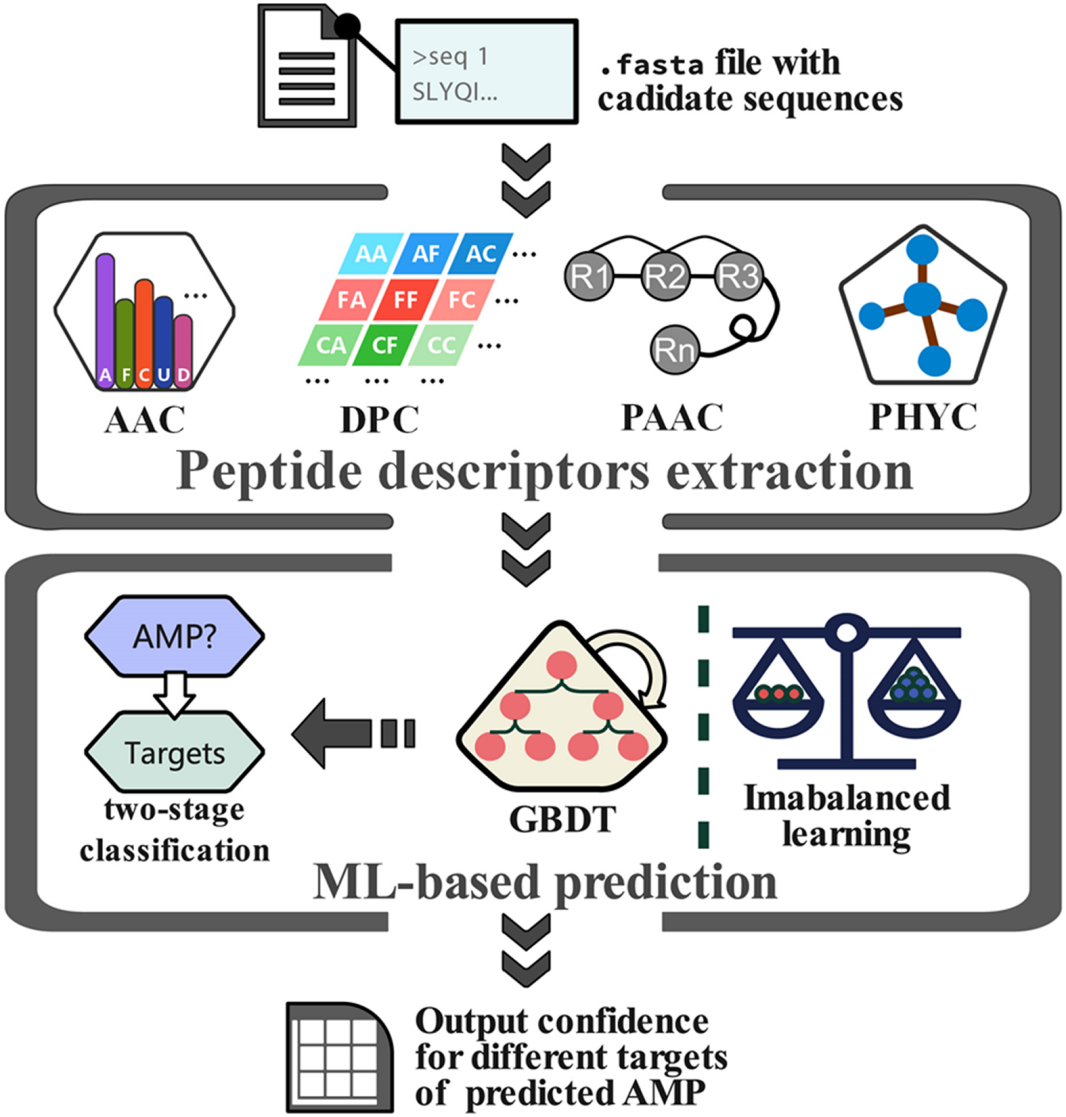
The schematic framework underlying AMP target prediction. First, the user-input sequences are transformed into one of four different groups of peptide descriptors including amino acid composition (AAC), dipeptide composition (DPC), pseudo-amino acid composition (PAAC) and physiochemical properties (PHYC). These descriptors are then used as the feature vector for processing during the two-stage classification process that relies on GBDT and imbalanced learning. This evaluation will then produce a confidence value (ranging from 0 to 1 as the potency level for targeting different microbes) for each of the predicted AMPs.

A previous iteration of this database used an alternative prediction tool to facilitate computer-aided AMP identification based on different species (51). It only focused on identifying general antimicrobial activity. However, the mechanisms of AMP targeting different microbes need to be emphasized. Thus, we proposed an enhanced prediction scheme for this version of dbAMP that uses a machine learning-based prediction model to identify specific targets based on the collected annotations of related AMP functional activities from the dbAMP. A schematic framework for this enhanced prediction is illustrated in Figure 4. We adopted a two-stage classification scheme (38,54) in which the first stage distinguished AMPs from regular peptides and the second stage was responsible for characterizing the specific function that targets different microbes, including bacteria (Gram-positive and Gram-negative, separately), viruses, fungi, and cancer and mammalian cells. This prediction scheme combines several peptide descriptors (55), which can encrypt the combinatorial and physicochemical properties of specific amino acids. The gradient boosting decision tree (GBDT) algorithm (56) was used at each of the tasks to establish the classifiers and the imbalanced learning strategies (57) were applied to improve the classifier’s performance by reducing the curse of insufficient positive labels within specific tasks. The prediction results are confidence values (ranging from 0 to 1) for each of the input peptides, which indicate the putative activities against different targets. Statistics about the predicted confidence value of training/test datasets are summarized in Supplementary Figure S2 and Supplementary Table S2. Users can conduct their screening process arbitrarily with the predicted confidence values, such as choosing the peptides with the largest confidences or directly discriminating with the default cutoff value (0.5). This prediction scheme achieved considerable performance in assisting with AMP design (Table 3). We also introduce our previously developed machine learning-based prediction scheme, AVPIden, for antivirus peptide target prediction (58). This method can characterize the specific targets of AVPs, including six different virus families and eight specific viruses, such as coronavirus. These prediction tools are provided on the ‘Analyze’ page.

**Table 3.** Prediction performance for the test dataset for each of the tasks in two-stage AMP prediction

Stage	Prediction task	SEN (%)	SPEC (%)	MeanACC (%)
First	AMP	93.34	91.37	92.36
Second	Anti-Gram-positive	91.14	88.42	89.78
	Anti-Gram-negative	89.58	88.79	89.19
	Antivirus	88.87	81.05	84.96
	Antifungal	93.86	57.92	75.89
	Anticancer	84.33	81.24	82.79
	Mammalian inhibition	78.49	79.08	78.79

TP, TN, FP and FN denote true positive, true negative, false positive and false negative, respectively. The sensitivity (SEN), specificity (SPEC) and mean accuracy (MeanACC) are defined as follows: SEN = TP/(TP + FN); SPEC = TN/(TN + FP); and MeanACC = 0.5(SEN + SPEC). The value 0.5 is a default cutoff of confidence values used to determine the positive or negative predictions.

## DISCUSSION

### New tools for identifying AMPs in large genomes

In this update, we launched a new integrated online tool designed to improve the prediction accuracy of small peptides (AMPfinder). This system uses the integrated AMP information in dbAMP to search for potential AMPs using genome/metagenome or proteome data and applied either via the dbAMP website or as a command-line tool. AMPfinder provides a preliminary annotation of the submitted DNA sequences based on the data available in dbAMP. AMPfinder can accept GenBank accession or GI numbers, pasted sequences or uploaded nucleotide sequence files in FASTA format. These files can contain more than one FASTA formatted sequence, such as whole genome sequencing assembly contigs or multiple proteins. AMPfinder analyzes these sequences and provides a detailed output of the predicted AMPs and source organism class. The dbAMP website also includes a new AMPfinder visualization tool for short peptide predictions in environmental samples using known AMP detection (Supplementary Figure S3A) and unknown AMP prediction and includes their source category (Supplementary Figure S3B). AMPfinder then provides preliminary annotations of AMP sequences based on the data available in dbAMP.

AMPfinder is the first package specifically designed for the identification of AMPs in large genomes. Previous software packages have also included tools for the prediction of AMPs. However, their main purpose was limited to plant species (52,53). Given this, we evaluate the accuracy of AMPfinder classifications using the representative *Periplaneta americana* (American cockroach) genome (ASM293952v1) as a test case. AMPfinder identified 11 highly homologous AMPs (Supplementary Table S3) in this genome when using the default search *E*-value threshold and model prediction values, which was consistent with previous studies (59). In addition, our prediction identified an additional 16 potential AMPs (Supplementary Table S4), highlighting the value of these tools.

### Collection of the coronavirus targeting peptides

**Figure 5.**
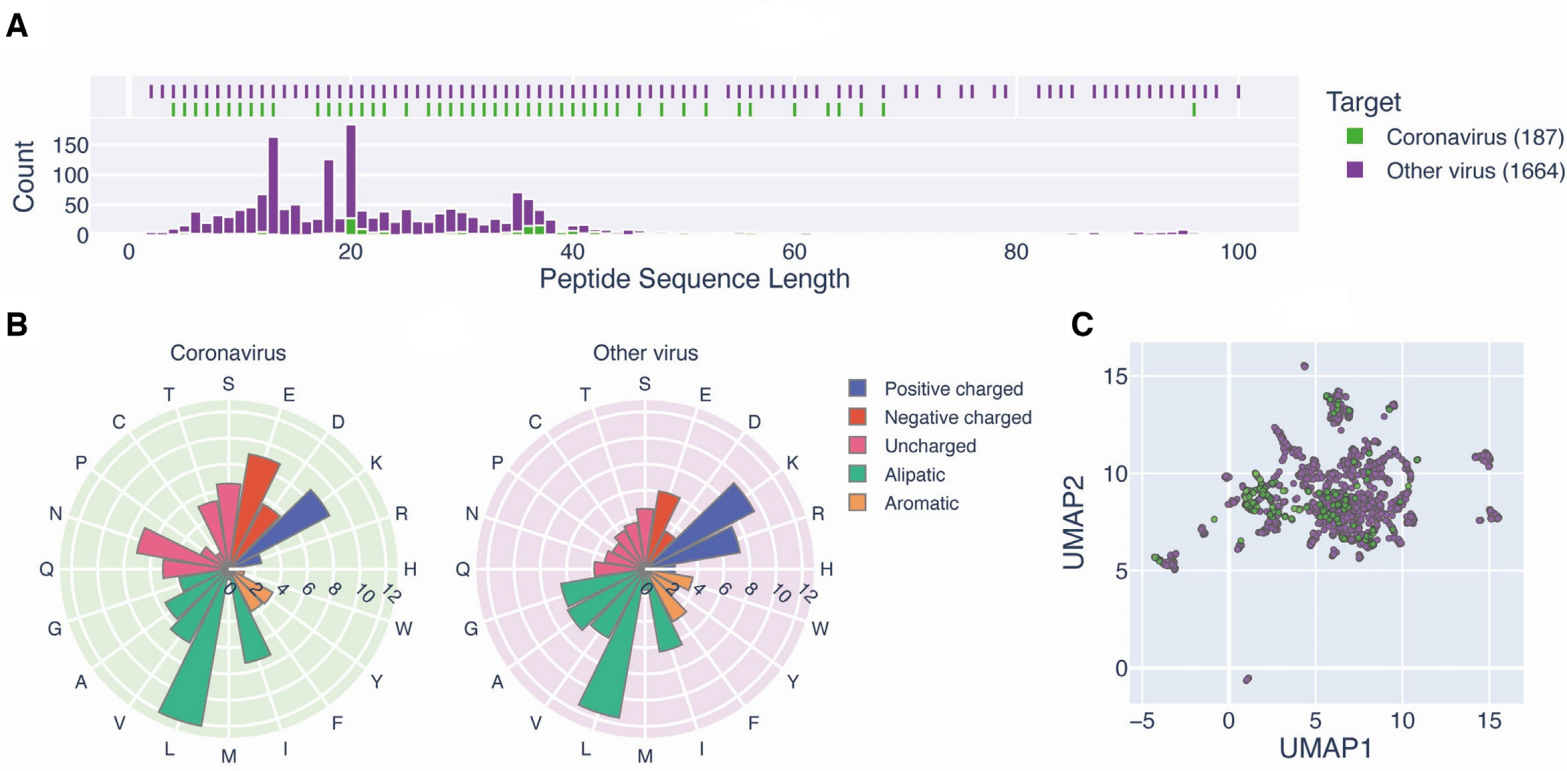
Summary of the properties of the peptides shown to target coronavirus and other viruses. (**A**) Length distribution of the anticoronavirus peptides (*n* = 187) and regular AVPs (*n* = 1664). To distinguish between AMPs and antimicrobial proteins, the entries with sequence length >100 amino acids (*n* = 1 for anticoronavirus peptides and *n* = 57 for other regular AVPs, respectively) are not shown in the histogram. (**B**) Average amino acid composition. Amino acids are categorized according to their physicochemical properties. (**C**) Dimension reduction of peptide sequences as extracted by tape, which reveals the differences between these peptides and where each point represents a peptide sequence with anticoronavirus (green) or regular antivirus (purple) activity.

It has been reported that several AVPs have shown some functional activity against coronavirus (6,60). Due to the current pandemic, the development of new therapeutic agents relies on prior knowledge of existing data, including existing computer-assisted methods to date (61). We collected a variety of anticoronavirus peptides from various resources and summarized their basic properties compared to normal AVPs. The length distribution (Figure 5A) of the regular antivirus peptides is slightly concentrated at <20 amino acids, but some of these AVPs can be longer than 100 residues. The mean value and actual distribution of these amino acids are shown in Figure 5B and Supplementary Figure S4, and the high frequency of positively charged and aliphatic amino acids in both categories of peptides may be related to their penetration of the viral membrane. Moreover, we computed the latent sequence encodings of these peptides using tape (62) and performed dimension reduction using UMAP (63) to inspect the differences between the anticoronavirus peptides and regular AVPs (Figure 5C). Although there is a clear distinction, the distance between the anticoronavirus peptides and regular AVPs remains small. This collection of anticoronavirus peptides may offer valuable information for the development of novel therapeutic agents against related pathogens.

## SUMMARY AND PERSPECTIVES

AMPs are promising candidates for resolving post-antibiotic effects, with an increasing number of studies suggesting that AMPs may act as potential therapeutic agents against various pathogens (64). Some studies have also suggested that some AMPs may inhibit COVID-19 (65). *In silico* peptide design can assist and accelerate the development process for novel AMPs (61,66), but relies on the related data stored in large databases and computer-aided analysis tools. Thus, dbAMP was established to provide a single platform combining any and all information on and computational analyses of novel AMPs. This revision of the dbAMP platform incorporates >28 000 unique AMP sequence entries from the literature and related databases with detailed annotations and computation-based physiochemical properties. We have improved the summary statistics of the entire database in an effort to provide more valuable perspective for researchers and established a novel 3D structure viewer for validated peptides and computationally derived putative structures for those without experimentally validated structures. This tool was designed to allow users to investigate the crucial mechanisms of AMP interaction with different pathogens. In addition, we integrated AMPfinder and AMP functional target prediction to produce a single platform solution for AMP development. The combination of these two analytical tools allows for the application of both proteome and genome data in the screening and identification of potential AMP sequences and provides their putative activity scores for a wide variety of pathogens. Eventually, we hope to maintain the dbAMP platform in real time to include cutting-edge developments and studies of novel therapeutic candidates and will try to establish a comprehensive encyclopedia of AMPs for scientific research.

## DATA AVAILABILITY

The dbAMP data content will be maintained and updated quarterly via the continuous survey of public resources and research articles. The database assistant system is now freely accessed online at http://awi.cuhk.edu.cn/dbAMP and all manually confirmed AMP resources can be accessed via the download page (https://awi.cuhk.edu.cn/dbAMP/download.php), allowing researchers to independently analyze our data. We also provide all previous versions of the database, which can be accessed through the ‘Previous Release’ page. Finally, the source code for AMPfinder can be accessed via https://github.com/BiOmicsLab/AMPfinder.

## Supplementary Material

gkab1080_Supplemental_File
