# Supplementary material for: dbAMP 2.0: updated resource for antimicrobial peptides with an enhanced scanning method for genomic and proteomic data
Source: Nucleic Acids Res. 2021 Nov 29;50(D1):D460–70. doi: 10.1093/nar/gkab1080 (PMC8690246; doi:10.1093/nar/gkab1080)
Supplement: gkab1080_Supplemental_File [file gkab1080_supplemental_file.docx]

**SUPPLEMENTARY MATERIALS**

**Figure S1. The distribution of AMP source organisms in dbAMP 2.0.**

**Figure S2. The distribution of predicted confidence scores among the training (top) and test (bottom) datasets under different prediction tasks of the AMP functional prediction.**

**Figure S3. Partial view of the *AMPfinder* alignment and prediction results page.**

**Figure S4. Strip plot illustrates the distribution of the amino acid composition’s difference between anti-coronavirus peptides and regular antivirus peptides.**

**Table S1. Performance of prediction datasets for the AMPs derived from different organisms.**

**Table S2. The summary statistics about the distribution of predicted confidence values (100% × (Mean ± Std)) among the train/test datasets for different prediction tasks.**

**Table S3. Homology detection of Antimicrobial Peptides (AMPs) from American Cockroach Transcripts.**

**Table S4. Putative prediction of Antimicrobial Peptides (AMPs) from American Cockroach Transcripts.**


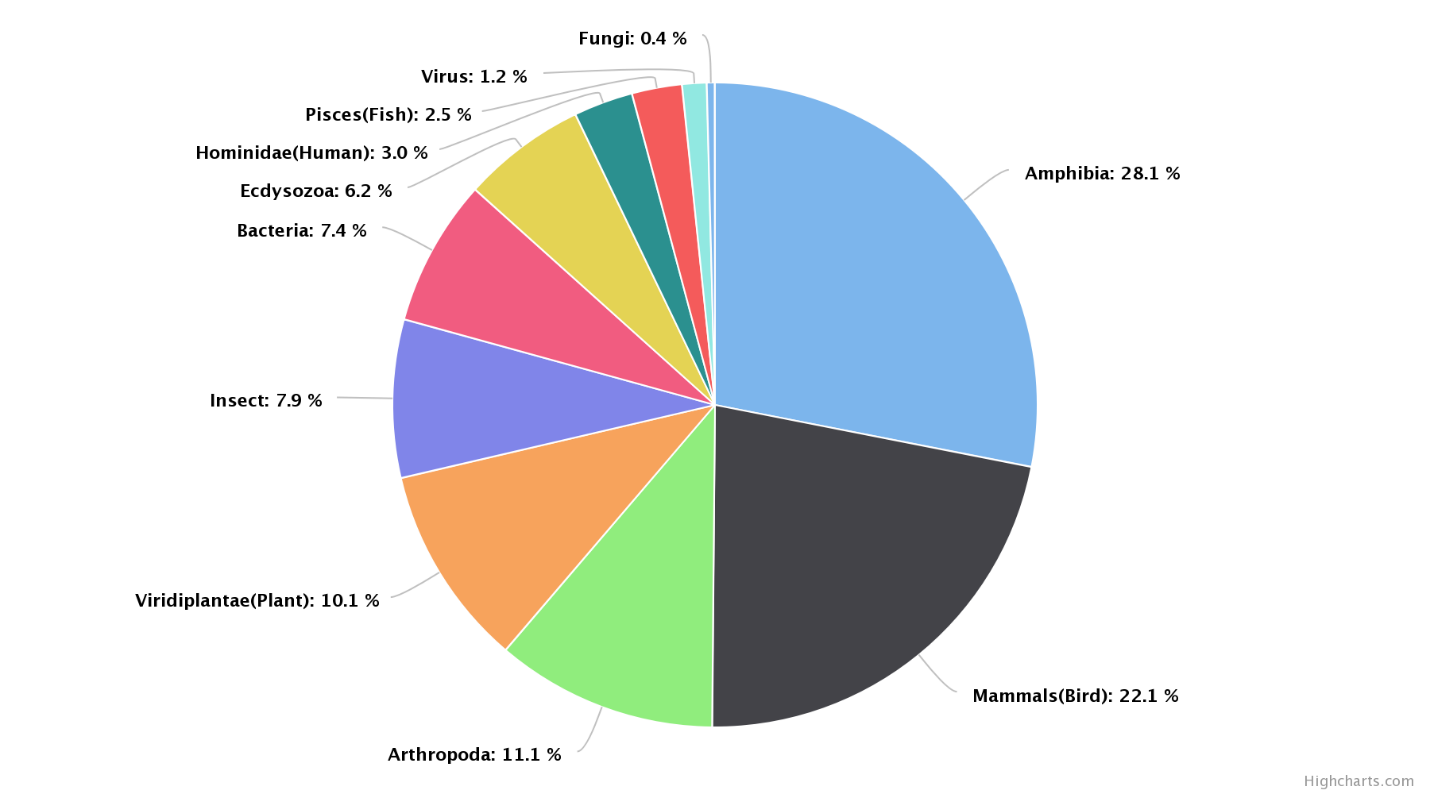


**Figure S1. The distribution of AMP source organisms in dbAMP 2.0.**

**
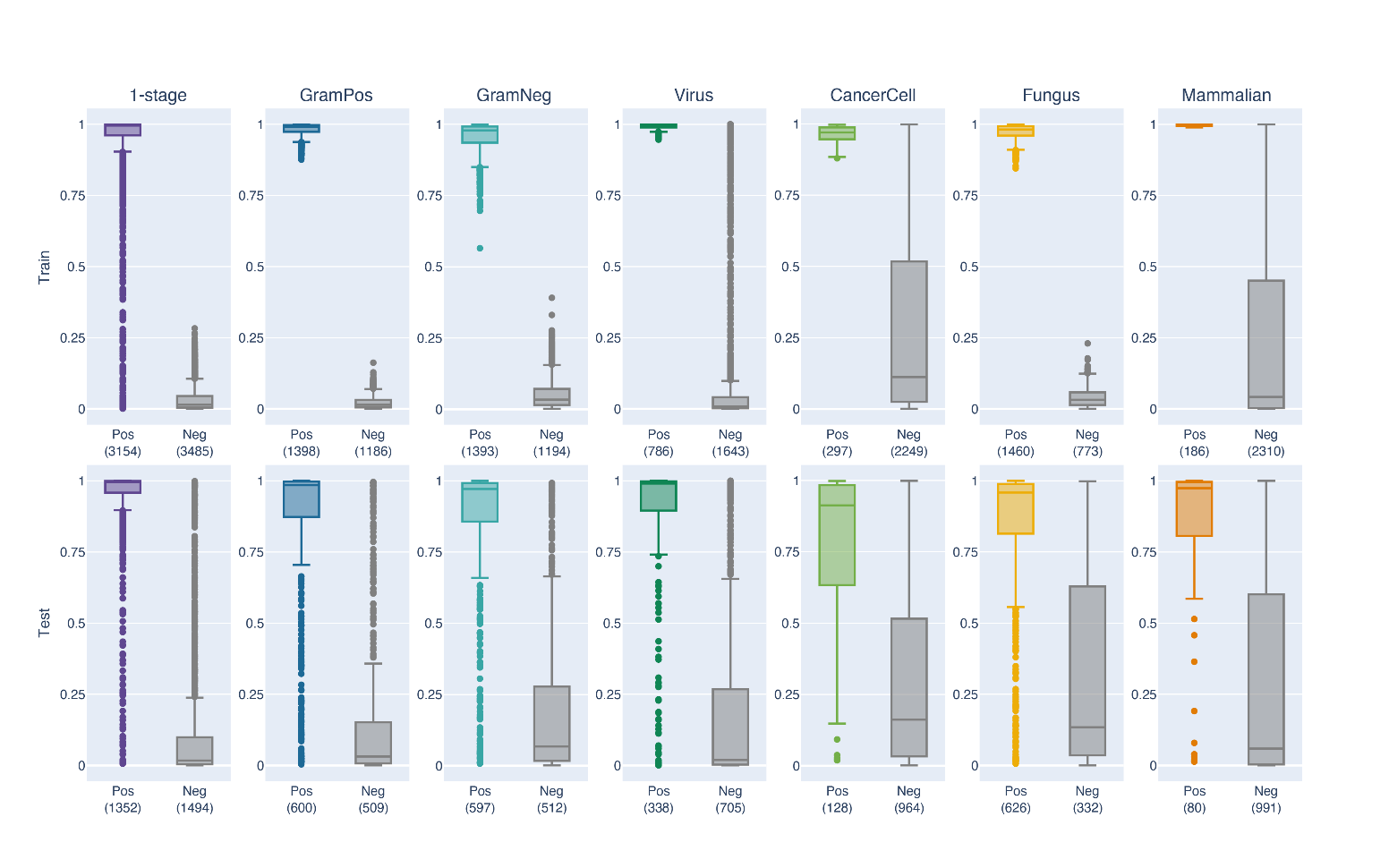
**

**Figure S2. The box-plotted distribution of predicted confidence scores among the training (top) and test (bottom) datasets under different prediction tasks of the AMP functional prediction.**


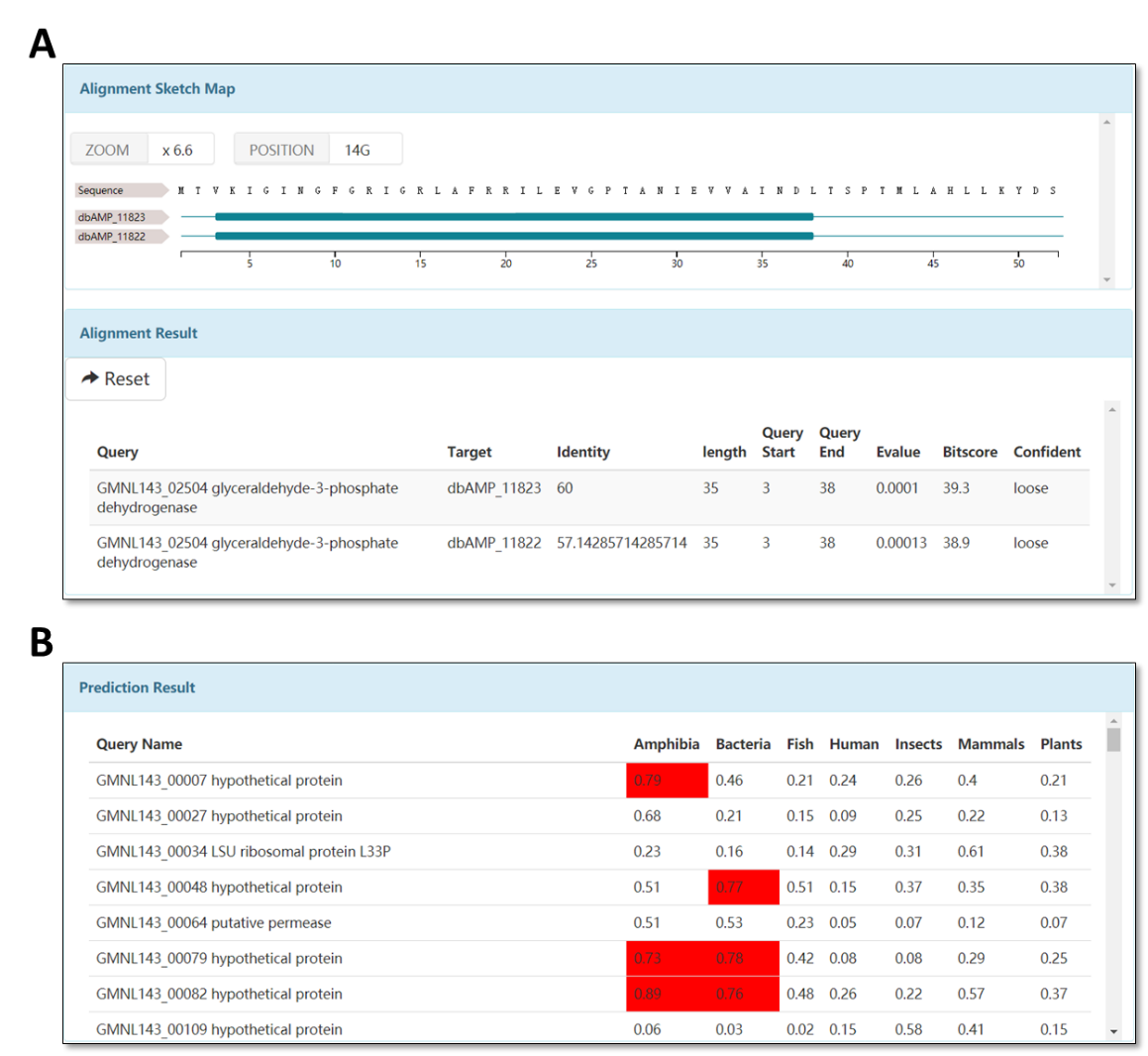


**Figure S3. Partial view of the *AMPfinder* alignment and prediction results page. (**A) Alignment result shows all records that aligned with sequences in dbAMP. Each set of records has a corresponding sketch map. (B) Prediction result lists all possible antimicrobial peptides. Each cell represents the predicted score. The red cells indicate that predicted score is greater than the set threshold in the home page.


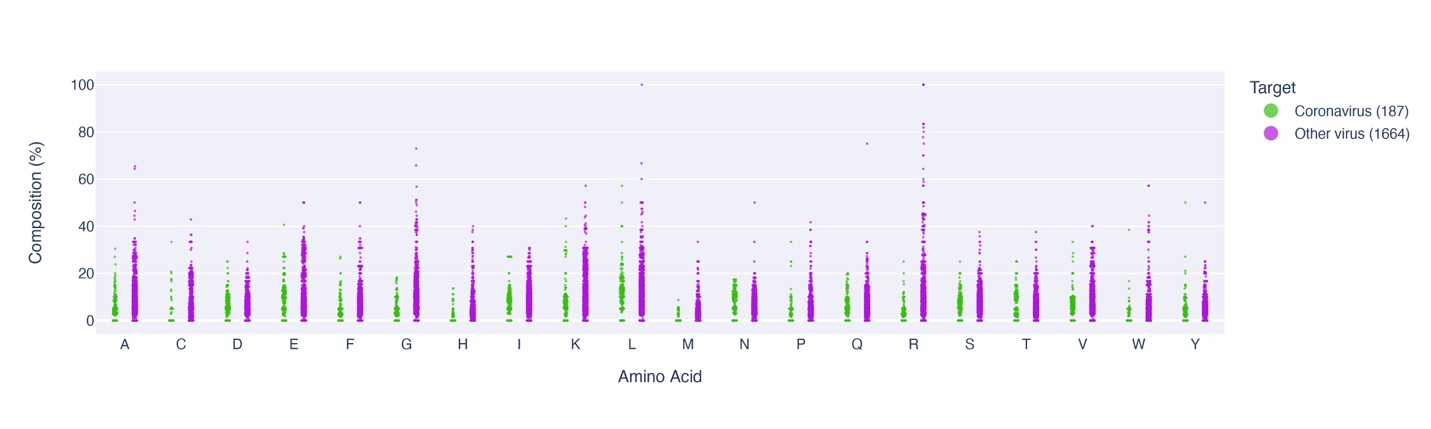


**Figure S4. Strip plot illustrates the distribution of the amino acid composition’s difference between anti-coronavirus peptides and regular antivirus peptides.**

**Table S1. Performance of prediction datasets for the AMPs derived from different organisms.**

| Organism | Training dataset | | | | Independent testing dataset | | | |
| --- | --- | --- | --- | --- | --- | --- | --- | --- |
|  | SN | SP | Acc | MCC | SN | SP | Acc | MCC |
| Amphibia | 99.19% | 99.18% | 99.19% | 98.10% | 100% | 98.24% | 98.80% | 97.30% |
| Bacteria | 95.94% | 96.18% | 96.16% | 73.50% | 96.51% | 96.36% | 96.36% | 74.60% |
| Human | 94.09% | 93.07% | 93.10% | 48.90% | 97.83% | 92.17% | 92.33% | 48.20% |
| Mammals | 94.42% | 95.24% | 95.19% | 70.80% | 92.79% | 94.56% | 94.46% | 67.30% |
| Fish | 96.84% | 96.87% | 96.87% | 78.90% | 100% | 97.00% | 97.18% | 81.00% |
| Insects | 96.36% | 96.33% | 96.34% | 83.80% | 100% | 97.56% | 97.82% | 90.00% |
| Plants | 97.53% | 97.39% | 97.39% | 82.20% | 97.78% | 97.94% | 97.93% | 85.10% |

SN = Sensitivity; SP = Specificity; Acc = Accuracy; MCC = Matthews Correlation Coefficient.

**Table S2. The summary statistics about the distribution of predicted confidence values (100% × (Mean ± Std)) among the train/test datasets for different prediction tasks.** The confidence values are separated according to whether they belong to positive data (+) or negative data (-).

|  |  | AMP | GramPos | AnGn | AnVi | AnCa | AnFu | AnMaml |
| --- | --- | --- | --- | --- | --- | --- | --- | --- |
| Train | (+) | 94.27 ± 2.29 | 98.16 ± 0.05 | 95.53 ± 0.28 | 99.26 ± 0.01 | 96.42 ± 0.07 | 97.29 ± 0.07 | 99.61 ± 0.00 |
|  | (-) | 3.33 ± 2.29 | 2.17 ± 0.05 | 5.21 ± 0.28 | 14.61 ± 0.01 | 28.62 ± 0.07 | 3.97 ± 0.07 | 25.52 ± 0.00 |
| Test | (+) | 94.01 ± 2.29 | 85.04 ± 0.05 | 85.08 ± 0.28 | 85.19 ± 0.01 | 78.76 ± 0.07 | 83.25 ± 0.07 | 82.84 ± 0.00 |
|  | (-) | 11.77 ± 2.29 | 16.41 ± 0.05 | 21.17 ± 0.28 | 21.22 ± 0.01 | 30.04 ± 0.07 | 31.63 ± 0.07 | 28.12 ± 0.00 |

**Table S3. Homology detection of Antimicrobial Peptides (AMPs) from American Cockroach Transcripts.**

| Sequence ID | dbAMP | Sequence | confidence |
| --- | --- | --- | --- |
| ISGCock_Contig04_0915 | dbAMP_04117 | ALQICTRNMIDDRLPYVADNVRPGTFIKQQRKQKQQRHHTSGTRKRMAKG | high |
| ISGCock_Contig13_4610 | dbAMP_12238 | HLYPCKLNLKLGKVPFHFLNLNHKGKSIMVNQQTCLYYIICQTR | high |
| ISGCock_Contig16_2060 | dbAMP_00571 | ISHNHLTAASITHVKNRGKYIYMHLKFRKTNVLI | high |
| ISGCock_Contig16_4974 | dbAMP_05151 | RKKVWFIFHVCPKLKQRILSDTHAKNKCRLSPLLIKSTKIKNET | high |
| ISGCock_Contig10_4736 | dbAMP_04157 | LMLCKGFLRHSYKSIHERGTKRGKLCRISRLALSSLP | high |
| ISGCock_Contig13_3006 | dbAMP_05997 | ANLLRHKVYGYCVLGPKGSSLGGIHGTWHDHHCSLIQRNPSTSTKGN | high |
| ISGCock_Contig05_0593 | dbAMP_08245 | MKTFLRLYRSLINKVLHV | high |
| ISGCock_Contig12_4176 | dbAMP_11756 | VVGRKHSILNCIPYLKKKKIMRLVESESIG | high |
| ISGCock_Contig15_1337 | dbAMP_04500 | KRMKLNAKKLSFCDHLNSYLNLSPTLFIHNSSKQWSHWLWHNGIRI | high |
| ISGCock_Contig15_1337 | dbAMP_10591 | KRMKLNAKKLSFCDHLNSYLNLSPTLFIHNSSKQWSHWLWHNGIRI | high |
| ISGCock_Contig07_3736 | dbAMP_04018 | CNYISFFRKCKNSQSTMYGCHRMNKCVFSSY | low |

The underlined peptide sequence is the result of blast alignment against to the dbAMP database.

**Table S4. Putative prediction of Antimicrobial Peptides (AMPs) from American Cockroach Transcripts.**

| AMPfinder | | | | PEPSTATS | | | CAMP | | | AMPA |
| --- | --- | --- | --- | --- | --- | --- | --- | --- | --- | --- |
| ID | Sequence | Class | Probability^a^ (Score >0.9) | length (≤ 50mer) | charge (>0(+)) | pI (8 ≤ pI ≤ 12) | Random Forest | SVM | Discriminant Analysis | Antimicrobial index^b^ (>=1) |
| ISGCock_Contig13_3331 | IRFGKFKNLRQKQENRCGDIFKQRQGLETCRHRLQFKIDLYISTNDK | AMP | 0.96 | 47 | 7.5 | 10.816 | AMP | AMP | AMP | 1 |
| ISGCock_Contig07_2123 | NIYHFFNINKTQFLLITHN | AMP | 0.98 | 19 | 2 | 9.303 | Non-AMP | Non-AMP | AMP | 1 |
| ISGCock_Contig13_1958 | KKKKKKLILRDLYNVVREFKKGYQARVKAIKDENDLLADS | AMP | 0.93 | 40 | 7 | 10.6901 | AMP | Non-AMP | AMP | 1 |
| ISGCock_Contig09_0640 | FIVTKNNNYHEHCSKFLYRLTRILVN | AMP | 0.92 | 26 | 4 | 9.9475 | Non-AMP | Non-AMP | AMP | 1 |
| ISGCock_Contig14_4969 | LKLSSLCHESIKLKEKNIIFFCI | AMP | 1 | 23 | 2.5 | 8.812 | AMP | AMP | AMP | 1 |
| ISGCock_Contig08_0798 | NKKQCSLIICGQFYRFCKAKF | AMP | 0.98 | 21 | 5 | 10.0685 | AMP | AMP | AMP | 1 |
| ISGCock_Contig14_3195 | VCKVAVRTLYCKKICFLVGDKVLTTSNYPFKLIKH | AMP | 0.96 | 35 | 6.5 | 10.1305 | AMP | AMP | AMP | 1 |
| ISGCock_Contig07_4712 | VFRVGLLPFPLPAFHHCSIKHHHNHVMLCSSPLMGHQLQCLW | AMP | 0.98 | 42 | 5.5 | 8.5493 | Non-AMP | Non-AMP | AMP | 1 |
| ISGCock_Contig13_0354 | FGIGKKFVCVCERCITEFLNTKSQLKNRKGIDLLFWKNREKNPKR | AMP | 0.93 | 45 | 8 | 10.7882 | AMP | AMP | AMP | 1 |
| ISGCock_Contig16_4259 | KRIRGEWRRIRLKSRTKYNAPVRDYKKILC | AMP | 0.99 | 30 | 10 | 11.8984 | AMP | AMP | AMP | 1 |
| ISGCock_Contig11_2637 | RNLVRNSLFSTKQEKYHLPCFIGILGIQCTRKKKEK | AMP | 0.92 | 36 | 7.5 | 10.8314 | AMP | AMP | AMP | 1 |
| ISGCock_Contig14_2165 | EILNSLKKVRSHIIFRHYDDIASRICAPLIGTII | AMP | 0.91 | 34 | 3 | 9.4058 | Non-AMP | Non-AMP | AMP | 1 |
| ISGCock_Contig06_1137 | EAAFMSASGKKQLVARRWVNNPVH | AMP | 0.95 | 24 | 3.5 | 11.6509 | AMP | Non-AMP | AMP | 1 |
| ISGCock_Contig05_0115 | NKINRKKRKSNYKHSYYHVINHQSSFIFLTL | AMP | 0.95 | 31 | 8.5 | 11.0608 | Non-AMP | Non-AMP | AMP | 1 |
| ISGCock_Contig04_1145 | IVLRSLFKNCRTGTHRADVPGKSASFILTELLCSLR | AMP | 0.93 | 36 | 4.5 | 10.7543 | AMP | AMP | AMP | 1 |
| ISGCock_Contig14_1205 | LIFIRRNCLCHTNSNRIGLGIYSSTAIIHHLINRLLDYPSLLCRKRIEY | AMP | 0.92 | 49 | 6.5 | 9.845 | AMP | AMP | AMP | 1 |

^a^ Probability score predicted by AMPfinder. ^b^ Antimicrobial index for each individual residue calculated by AMPA.
